# Seizures worsen tau spread preferentially in hyperactive neurons in a murine tauopathy model

**DOI:** 10.1002/alz70861_108774

**Published:** 2025-12-23

**Authors:** Aaron J Barbour, Keegan Hoag, Virginia M. M.‐Y. Lee, Delia M Talos, Frances E Jensen

**Affiliations:** ^1^ University of Pennsylvania, Philadelphia, PA USA

## Abstract

**Background:**

The spread of pathological tau through communicating neurons is central to neurodegeneration in Alzheimer’s disease (AD). In addition, seizures are a common occurrence in AD and exacerbate both cognitive and pathological disease outcomes. We have recently demonstrated increased seizure susceptibility, and enhanced seizure‐induced tau spread in a β‐amyloid mouse model. We next sought to determine whether there are similar seizure susceptibility phenotypes and brain‐wide effects of seizure induction on tau spread in a tauopathy mouse model. Given the role of tau in network excitability, we hypothesized that T40PL‐GFP mice, harboring a GFP tagged pathogenic MAPT mutation (P301L), would show increased seizure susceptibility, and that seizures would worsen tau spread in these mice.

**Method:**

To investigate seizure‐tau interactions, we crossed the T40PL‐GFP mouse model with targeted recombination in active populations mice (TRAP; T40/WT‐TRAP) to permanently label seizure activated neurons with tdTomato. To induce tau spread, we unilaterally injected human AD brain derived tau lysate (AD‐tau) into the right hippocampus and cortex at three months of age. Seizures were induced with pentylenetetrazol (PTZ) kindling (or control, saline protocol) 2‐3 weeks following AD‐tau injection and seizure‐activated neurons were labelled with tdTomato on the final PTZ administration via 4‐hydroxytamoxifen administration. All mice were euthanized three months following AD‐tau seeding, and intact T40‐TRAP brains underwent clearing, immunolabeling for NeuN, and light sheet microscopy to image tau‐GFP, tdTomato, and NeuN. Whole‐brain datasets were registered to the Allen Brain Atlas and tdTomato+ and tdTomato‐ (NeuN+) somatic tau‐GFP were mapped.

**Result:**

We found that T40PL‐GFP mice had increased seizure susceptibility compared to WT mice (*n* =9‐11, *p* <0.05). We also found significantly increased tau‐GFP in seizure activated tdTomato+ neurons compared to neurons in saline treated mice in the ipsilateral and contralateral (to injection site) striatum, ipsilateral perirhinal and orbital areas, and contralateral nucleus raphe (*n* =4‐5, *p* <0.05). tdTomato+ neurons also had elevated tau‐GFP compared to the surrounding tdTomato‐ neurons in the ipsilateral striatum and perirhinal area (*n* =4‐5, *p* <0.05).

**Conclusion:**

Together, these results demonstrate a bidirectional relationship between neuronal hyperactivity and tau pathology, and suggest seizures and seizure activated neurons as targets to slow progression of tau pathology.

